# Ganciclovir Antiviral Therapy in Advanced Idiopathic Pulmonary Fibrosis: An Open Pilot Study

**DOI:** 10.1155/2011/240805

**Published:** 2011-04-07

**Authors:** J. J. Egan, H. I. Adamali, S. S. Lok, J. P. Stewart, A. A. Woodcock

**Affiliations:** ^1^Advanced Lung Disease and Lung Transplant Programme, Department of Respiratory Medicine, Mater Misericordiae University Hospital, University College Dublin, Eccles Street, Dublin 7, Ireland; ^2^Respiratory Research Group, University of Manchester, Manchester Academic Health Sciences Centre, UK; ^3^Division of Medical Microbiology and Genitourinary Medicine, University of Liverpool, UK; ^4^NIHR Translational Research Facility in Respiratory Medicine, University Hospital of South Manchester, UK

## Abstract

*Hypothesis*. Repeated epithelial cell injury secondary to viruses such as Epstein Barr and subsequent dysfunctional repair may be central to the pathogenesis of IPF. In this observational study, we evaluated whether a combination of standard and anti-viral therapy might have an impact on disease progression. *Methods*. Advanced IPF patients who failed standard therapy and had serological evidence of previous EBV, received ganciclovir (iv) at 5 mg/kg twice daily. Forced vital capacity (FVC), shuttle walk test, DTPA scan and prednisolone dose were measured before and 8 weeks post-treatment. *Results*. Fourteen patients were included. After ganciclovir, eight patients showed improvement in FVC and six deteriorated. The median reduction of prednisolone dose was 7.5 mg (44%). Nine patients were classified “responders” of whom four showed an improvement in all four criteria, while three of the five “non-responders” showed no response in any of the criteria. Responders showed reduction in prednisolone dosage (*P* = .02) and improved DTPA clearance (*P* = .001). *Conclusion*. This audit outcome suggests that 2-week course of ganciclovir (iv) may attenuate disease progression in a subgroup of advanced IPF patients. These observations do not suggest that anti-viral treatment is a substitute for the standard care, however, suggests the need to explore the efficacy of ganciclovir as adjunctive therapy in IPF.

## 1. Introduction

Idiopathic pulmonary fibrosis (IPF) is an increasingly common disease with a poor prognosis. Standard therapy has little or no impact in patients with advanced disease. The emerging hypothesis for the pathogenesis of IPF is of repeated epithelial cell injury leading to fibrogenesis and dysregulated repair [1]. The cause of repeated epithelial cell injury has received little attention. 

Herpesviruses provide a potential source of repeated epithelial cell injury. A role for viruses, especially for Epstein-Barr virus (EBV) in the pathogenesis of IPF has been suggested by serological and tissue based studies [2–4]. We have previously demonstrated the presence of EBV DNA in surgical samples taken from IPF patients [4]. EBV antigens have been localised by immunohistochemistry to type II alveolar cells [4]. Animal models have showed that a Herpesvirus can act as a cofactor in the development of pulmonary fibrosis [5]. Mora and colleagues showed that Herpesvirus induced fibrosis in a conditioned host and removal of a viral injury attenuated lung fibrosis [6]. 

 We hypothesised that a combination of standard treatment and antiviral therapy may have an impact on disease progression in IPF. The aim of this pilot study was to determine the efficacy of intravenous (iv) Ganciclovir in a subgroup of advanced IPF patients unresponsive to standard therapy. 

## 2. Methods

### 2.1. Patients

Between 1996 and 2000 we studied patients with advanced progressive IPF patients who were progressively deteriorating in spite of standard therapy, including Prednisolone, Azathioprine and Cyclosporin. All patients had evidence of previous EBV infection based on EBV-IgG serology detected using indirect immunofluorescence. All patients had finger clubbing, late inspiratory crackles, and restrictive lung function studies. The diagnosis of IPF was supported by a typical HRCT appearance (*n* = 14) and/or diagnostic lung biopsy (*n* = 3) [7]. Blinded histological review confirmed the presence of usual interstitial pneumonia.

### 2.2. Treatment

Patients were offered open label treatment with Ganciclovir (Cytovene Roche Pharmaceuticals, Basel Switzerland) given intravenously at 5 mg/kg twice daily for a period of 2 weeks.

### 2.3. Clinical Measures

Before and 8 weeks after treatment, lung function, exercise tolerance, DTPA clearance and prednisolone dosage were measured as four key clinical response markers. An improvement in three of these four parameters was considered to represent a positive therapeutic response and this patient was defined as a “responder”.

**Lung Function**

Spirometry best of 3 efforts within 5% (Gould wet spirometer) was measured. Calibration of the equipment was performed on a daily basis prior to the commencement of the procedure. A minimum of three values with less than 5% variability was required; the best response was recorded. Carbon monoxide lung transfer factor (TLCO, corrected for haemoglobin) and coefficient (KCO) were measured according to ATS guidelines [7] and expressed as percentages of the predicted values calculated according to sex, weight, and age.

**Exercise Tolerance**

This was assessed by the shuttle walk test, validated previously in IPF patients [8]. In brief, this involved the patient walking a 10 m course at increasing speed. Marker cones positioned 0.5 m from each end avoided the need for abrupt change in direction. The walking speed, which steadily increased, was controlled by audio signals played from an audio player. The test was terminated when (a) the patient was unable to maintain the required speed due to breathlessness (b) the operator deemed the patient had failed to complete a shuttle in the time allowed (more than 0.5 m away from the cone when the bleep sounded) or (c) the patient attained 85% of the predicted maximal heart rate.

**DTPA Scan**

^99m^Tc-DTPA was aerosolised using pressurised air (Aerotech II nebuliser, CIS-US, Bedford, MA, USA). Patients were seated in front of a gamma camera while inhaling the ^99m^Tc-DTPA through a mouthpiece during tidal breathing. The mouthpiece contained a one-way valve and the patients wore a nose clip. Inhalation continued for 5 min or until a suitable count rate was detected over the lungs. Images were then taken for 60 minutes by a gamma camera with a general-purpose collimator (Starcam General Electrics Medical Systems, USA). Images were acquired at an image resolution of 64 × 64 using frame duration of 20 seconds for at least the first 15 minutes. Regions of interest in both lungs were acquired and subtraction of background activity was performed through identification of a region between the kidneys or in the shoulders. The rate of radionucleotide disappearance from the lung was used to calculate solute clearance. Clearance rates from each patient referred to both monoexponential and biexponential clearance analysis. Curve stripping was used to separate the two components of the biexponential disappearance curves. The contribution of the slow component to the early part of the curve was calculated and subtracted out, isolating the fast component.

Four DTPA variables were considered. These were half times for the fast component and the slow component of the biexponential curve, the fraction of the tracer cleared by the fast component and the half time from a monoexponential approximation to the early part of the curve. The half time from a monoexponential approximation to the first 15 minutes of the curve was also calculated. The normal half time value for this parameter was greater than 45 minutes. An exponential fit was derived from the first 15 minutes of the curve and the half time for clearance of the DTPA from the lungs was determined [9–11].

**Prednisolone Dose**

The ability to reduce Prednisolone in 2.5 mg steps was based upon the patients' symptoms and lung function.

A lung function technician and radiologists who were blind to the patients underlying therapy or clinical condition performed the assessment of lung function, exercise tolerance and DTPA clearance.

### 2.4. Statistical Analysis

This was an exploratory study and no formal power calculation was made. The data were presented as means and comparisons made using paired *t*-tests. A value of *P* < .05 was accepted as statistically significant. 

## 3. Results

Fourteen patients (5 males, 9 females mean age 58 years (41–73) were offered therapy based on disease progression and EBV seropositivity (Table 1). 

**Figure 1:**
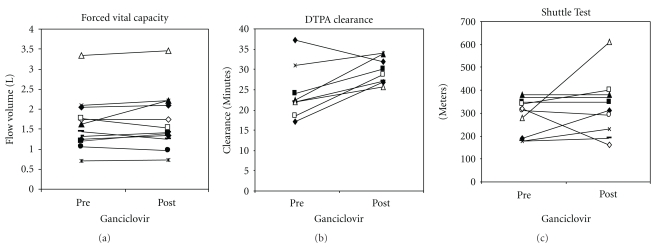
Patient characteristics following Ganciclovir. *Some patients were unable to perform the test due to breathlessness.

All patients showed a restrictive defect (mean FEV_1_/FVC ratio 86%) with reduction in lung volumes (mean FVC 1.73 L (52% predicted) (range 0.71–3.35 L), TLC at 59% predicted (range 2.1–4.1 L)) and an impairment of transfer factor (mean DLCO 3.42 ml/min/mmHg (44% predicted), range 1.22–5.6) (Figure 1).

Nine of the 14 patients were classified as “responders” (mean age 56.3 years (41–73), 2 male) with four showing an improvement in all four criteria. In the non-responders group (*n* = 5, mean age 61 years (range 57–66 years), 2 female) three of the five showed no response in any of the parameters used. Although females appeared more likely to respond, a chi-square test did not suggest a gender difference (*P* = .2). 

Responders had a significant reduction in Prednisolone dosage and improvement in DTPA clearance (*P* = .02 and *P* = .001, resp.) (Figure 1). Responders tended to be younger than non-responders (Mean age 56.3 years versus 60.8 years, *P* = .21). No significant adverse effects were observed from the therapy. 

 Following treatment the patients were subject to a follow-up, for up to 12 months. Of the nine responders, five underwent lung transplantation and six died. In the non-responder group two received lung transplantation and three had died at the end of the follow-up period (see Table 2).

## 4. Discussion

The long-term prognosis of progressive IPF is very poor, as is clear in this cohort of patients. This observational report suggests that a 2-week course of intravenous Ganciclovir may temporarily attenuate disease progression in a subgroup of patients with advanced IPF. This adds to the emerging evidence that a viral cofactor may contribute to disease progression in some patients, and provides a framework for the planning of a randomised treatment study.

Vergnon and colleagues first implicated EBV in the aetiology of IPF when serological testing identified elevated levels of IgG and IgA against viral capsid antigen (VCA) in these patients [2]. Studies utilising both immunohistochemistry and PCR have confirmed this association. Subsequently Tsukamoto and colleagues demonstrated that the presence of EBV latent membrane protein in the lung tissue of IPF patients was associated with rapid disease progression [12]. Animal model studies suggest that EBV alone does not cause pulmonary fibrosis but it may represent a cofactor in disease progression or act as a source of injury in a predisposed host [5]. In IFN-gamma receptor-deficient mice, Herpes viral infection led to pulmonary fibrosis [6]. These authors further showed that antiviral therapy controlled virus replication during chronic infection and prevented lung fibrosis [6].

Our cohort included patients with progressive IPF with a mean DLCO of 40% predicted and an overall survival of 8 months. Six patients died and 5 underwent transplant. Therefore, a dramatic response to treatment should not be anticipated. However, the short-term impact of Ganciclovir was encouraging in improving surrogate markers of disease progression. As expected the clinical response seen in the nine responders was modest. Treatment studies in advanced IPF are difficult because specific measures of disease severity or short-term disease progression are limited. This is a particular problem in an open observational study. In an attempt to overcome this problem we chose 4 surrogate markers (vital capacity, DTPA clearance, exercise tolerance and prednisolone dosage). Lung function, DTPA scans and exercise capacity parameters have previously been used individually as markers of disease activity and prognosis [11]. The shuttle walk test was chosen as the exercise test because it has previously been shown to correlate with VO_2_max [8]. This test in contrast to the six-minute walk test is less affected by the subjective efforts, which is likely to occur in a study of short duration as presented here. Each parameter has its individual merit, although no one test is ideal. We believe that the combination of these tests results in an improved objective overview of the response. Similarly, we did not measure serial DLCO because of variability and the inability of patients to perform the tests at the requested time due to severity of their illness. 

Tissue based studies suggest that 50% of IPF patients have EBV [3]. More recently it has been shown that Herpesvirus DNA is consistently found in lung tissue from patients with IPF and that EBV and human Herpes virus 8 show viral antigen primarily in the airway epithelial cells [13]. One difficulty in interpreting our data is absence of a surrogate marker of tissue specific viral activity. The patients were too unwell to undergo further biopsies and therefore treatment had to be empirical. Patients were selected on the basis of having positive serological (IgG) testing for EBV. 

King and colleagues have correlated the fibroblastic foci observed in IPF biopsies as a survival marker [1]. The persistent epithelial damage and unrelenting proliferation of fibroblasts need to be targeted in order to arrest the progressive disease and fatal outcomes associated with IPF [1]. EBV infection has been shown to persist in patients with IPF and may contribute to epithelial damage; its role in the pathogenesis of IPF requires closer scrutiny. The short course of intravenous Ganciclovir in patients with advanced disease in this study may not have allowed sufficient time to produce more significant results. Future studies should focus on oral treatment in a cohort of patients with less advanced disease. 

This study provides interesting preliminary data. However, to progress the thesis, a study including controlled group data would be required to strengthen the significance of the observations presented. Observation studies as presented are prone to bias in patient selection. It is also uncertain whether the duration of therapy chosen was optimal. A 2 week course of intravenous Ganciclovir was chosen for practical reasons. It is possible that a prolonged course of oral therapy may have a more beneficial effect. 

 A single short course of treatment with intravenous Ganciclovir produced a temporary benefit in a subgroup of patients with progressive and treatment-resistant IPF patients. We emphasise that our findings do not suggest that antiviral therapy is a panacea or substitute for standard care. The observations are interesting in that the data suggests that a viral cofactor may contribute to disease progression and provide a basis for the planning of a randomised controlled study. It is possible that maintenance oral valganciclovir might maintain any benefit arising from intravenous Ganciclovir.

##  Conflict of Interests 

The authors declare no conflict of interests.

## Figures and Tables

**Table 1:** Patient demographics.

Patient	Sex	Age	Method of diagnosis*	Forced vital capacity (L)	Total lung capacity (L)	DLCO (mL/min/mmHg)	Response to treatment	Outcome (after treatment)
1	M	66	Biopsy/HRCT	2.05	3.69	3.07	Responder	Dead (9 months)
2	F	51	HRCT	1.21	2.11	^#^	Responder	Alive
3	F	41	Biopsy/HRCT	1.63	3.3	3.93	Responder	Alive
4	F	71	HRCT	2.09	3.59	5.61	Responder	Dead (30 mths)
5	F	62	HRCT*	0.71	^#^	^#^	Responder	Transplanted (2 mths)
6	M	60	HRCT	2.52	4.18	4.3	Nonresponder	Dead (5 mths)
7	F	66	HRCT	1.07	2.89	2.94	Nonresponder	Dead (7 mths)
8	F	60	HRCT	1.75	3.02	1.39	Nonresponder	Dead (6 mths)
9	M	57	HRCT*	1.72	2.99	2.58	Nonresponder	Transplanted (4 mths)
10	F	50	HRCT*	1.43	2.59	^#^	Responder	Transplanted (4 mths)
11	F	46	HRCT*	1.25	2.39	3.9	Responder	Transplanted (11 mths)
12	M	61	HRCT*	1.88	3.86	3.3	Nonresponder	Transplanted (2 mths)
13	F	73	HRCT	1.60	3.17	3.2	Responder	Dead (9 mths)
14	M	47	Biopsy/HRCT	3.35	^#^	^#^	Responder	Alive

*All patients had a diagnostic HRCT. Three patients had diagnostic histology concurrently by VATS (video assisted thoracoscopy) and a further five patients had the histological confirmation confirmed in the explanted lung after transplantation.

^#^Patients were unable to perform testing.

**Table 2:** Summary of the non-responders (*n* = 5) and responders (*n* = 9) before and after Ganciclovir. Comparison by paired *t*-test.

Parameter	Responders			Nonresponders		
	Pre	Post	*P* value	Pre	Post	*P* value
FVC (litres)	1.70	1.85	.046	1.76	1.37	.08
DTPA (minutes)	22.4	28.2	.001	25.2^#^	17*	^@^
Shuttle (meters)	286	307	.08	260	273	.52
Prednisolone (mg)	19	10	.02	21	13.5	.95

*3 of the 5 patients were unable to perform test.

^#^1 of the 4 patients was unable to perform test.

^@^No paired samples available for statistical analysis.
